# Treatment of Phthiriasis Palpebrarum and Crab Louse: Petrolatum Jelly and 1% Permethrin Shampoo

**DOI:** 10.1155/2015/287906

**Published:** 2015-09-15

**Authors:** Yunus Karabela, Gurkan Yardimci, Isik Yildirim, Eray Atalay, Semsi Nur Karabela

**Affiliations:** ^1^Department of Ophthalmology, Esenler Hospital, Istanbul Medipol University, Esenler, 34230 Istanbul, Turkey; ^2^Department of Dermatology, Esenler Hospital, Istanbul Medipol University, Esenler, 34230 Istanbul, Turkey; ^3^Department of Clinical Microbiology and Infection Disease, Bakirkoy Dr. Sadi Konuk Training & Research Hospital, Bakirkoy, 34147 Istanbul, Turkey

## Abstract

Phthiriasis palpebrarum is an uncommon cause of blepharoconjunctivitis in which Pthirus pubis infest the eyelashes. We report a case of unilateral phthiriasis palpebrarum with crab louse. A 45-year-old man presented with conjunctival hyperaemia and moderate itching associated with irritation, and crusty excretions of the eyelashes in the left eye. Careful slit-lamp examination revealed many lice and nits in left eye and mild conjunctival hyperaemia. No abnormality was found in the right eye. On dermatologic examination, only one louse was found at the pubic area. The patient was treated effectively with petrolatum jelly (Vaseline) and 1% permethrin shampoo (Kwellada 1% shampoo). At the end of the first week no louse or nit was present on eyelashes and pubic area.

## 1. Introduction

Three types of lice infest humans, feed on blood, and reproduce on the body: the body louse (*Pediculus humanus corporis*), the head louse (*Pediculus humanus capitis*), and the pubic louse (crab louse). Phthiriasis palpebrarum (ciliary phthiriasis), caused by* Pthirus pubis*, is an uncommon cause of blepharoconjunctivitis; therefore, this condition is easily misdiagnosed. When it occurs, genital involvement must be suspected [1–4]. We report a case of unilateral phthiriasis palpebrarum accompanied by lice and nits on the eyelashes and one louse on the pubic hairs area together.

## 2. Case Report

**Figure 1:**
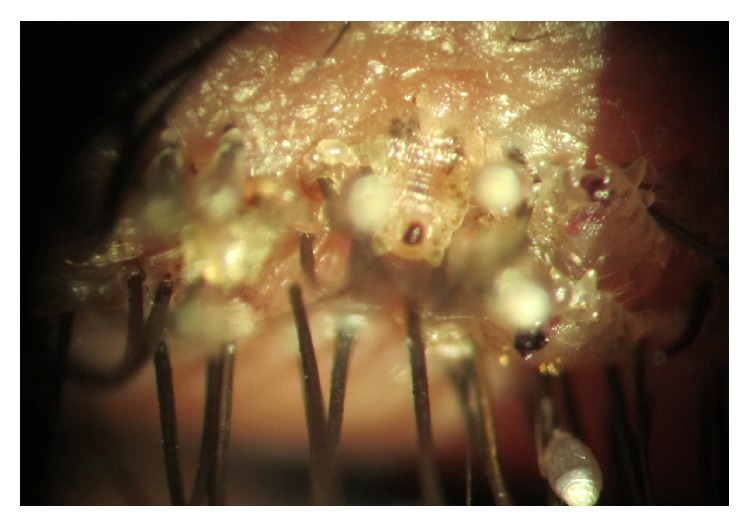
Slit-lamp photo; multiple lice and nits anchored at the eyelashes (pretreatment).

**Figure 2:**
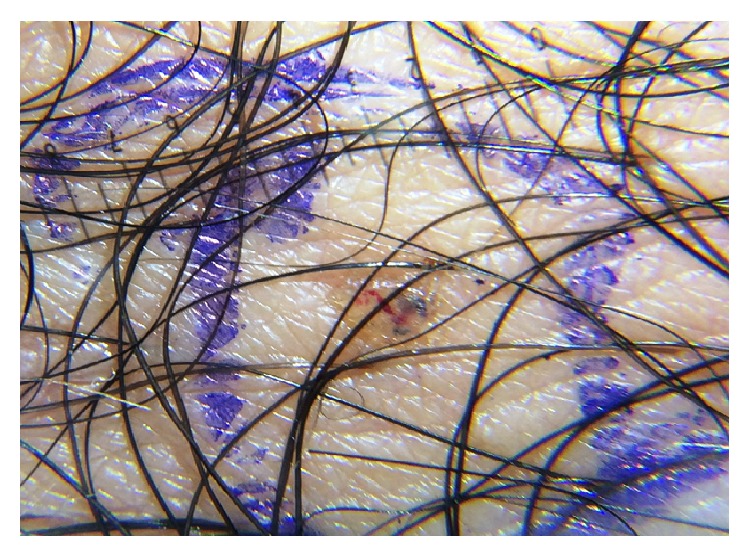
Photo; a louse in the pubic hair area (pretreatment).

**Figure 3:**
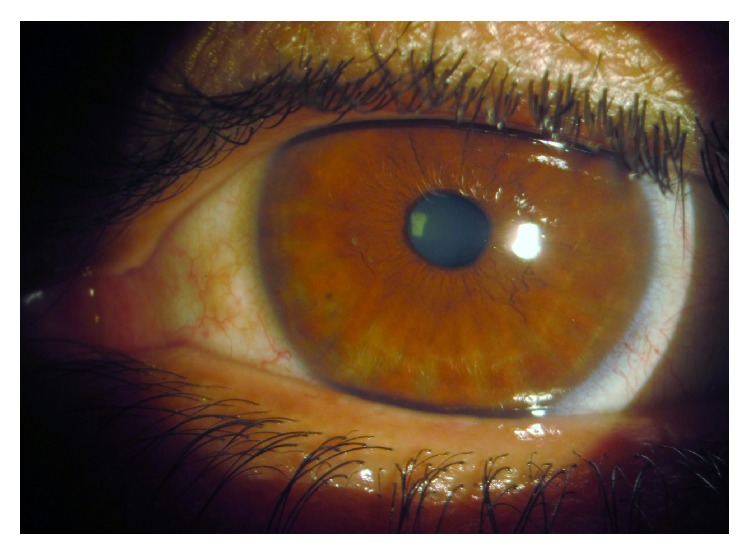
Slit-lamp photo; no louse and nit (posttreatment, 1st week).

A 45-year-old man was admitted to our ophthalmic department with a week history of itching, tearing, redness, irritation, and crusting in left eye. He had no specific ocular or systemic disease. His visual acuity was 20/20 in both eyes. A slit-lamp examination showed reddened, crusty eyelid margins and mild conjunctiva hyperaemia. Multiple, mobile semitransparent lice and translucent nits were detected at the left upper lid lashes (Figure 1). There was no abnormal finding in the cornea. No lice or nits were detected in the fellow eye. He was referred to the Department of Dermatology to exclude lice infestation of other body areas. On dermatologic examination, only one louse was detected in the pubic area (Figure 2), but the other areas of body were clear. He had past history of multiple heterosexual contacts. The search of sexually transmitted diseases was negative. He was treated with petrolatum jelly applied thickly to the lid, 2 times daily for about 2 hours. After waiting for about 2 hours, 1% permethrin shampoo (Kwellada, Ali Raif, and Turkey) was applied to the eyelid for 10 minutes and washed off, leading to elimination of the lice and nits. Similar treatment was given for the crab louse. The patient was advised to wash all clothing and fomites. We explained that his partners should be treated as well, and the patient was warned of the venereal disease. All adult lice on the left eyelash and pubic area were eradicated within 3 days. There were only a few nits clipping on the left eyelash after three-day application. No louse was found at the pubic area. Slit-lamp examination was performed a week later, no louse or nit was seen (Figure 3), and the treatment was stopped. No side effects due to the treatment were detected. Follow-up at one month revealed no recurrence of the condition.

## 3. Discussion

There are three species of blood sucking lice, small, tiny, and wingless insects, found on human [2–5]. Morphologically head and body lice are very much alike; however, body lice are slightly larger. These lice have abdomens being longer rather than being broad and their six legs are equal in size. In contrast, the abdomen of the crab louse is about as wide as or even slightly wider than its length and the second and third pairs of legs (its distinctive crablike appearance) are thicker than the first pair. Crab lice are much smaller (1 to 2 mm) than head and body lice. It is a slow-motion organism [1, 3, 6].

The adult female lays 3 eggs per day and 26 eggs per lifetime, which hatch every 7 to 10 days [7]. The eggs are visible to the unaided eye as 0,5 mm long oval shaped brownish opalescent pearls. The incubation period is 1 week [1, 4, 7, 8].

Adult pubic lice infest hairs of the scalp, axilla, chest, thighs, pubic area, and, rarely, eyebrows and eyelashes. The affliction of eyelashes is rare but when it occurs, it is usually due to the crab louse and rarely due to the head lice. The body louse is never found in eyelashes [3, 4, 8–10]. However, isolated palpebral involvement has been described [2, 4, 5, 11–13].

Pubic lice are able to easily ambulate and travel from groin to eyelashes or other hairy areas by themselves. However, in adults, they are commonly transferred from groin area to eyes by hands or less commonly by infected clothing or bed linen. In children, eyelashes are the most common site of the infestation [10, 14] and phthiriasis palpebrarum may be a sign of sexual abuse, and the possibility of abuse should be investigated. Transmission by bed linens and infested clothing is less likely and doubted by some [7, 10, 14, 15].

The symptoms associated with phthiriasis palpebrarum range from pruritic lid margin to blepharitis with marked conjunctival inflammation [5]. It may be confused with other forms of blepharitis, especially seborrheic blepharitis, unless one looks for the above signs. Diagnosis can be made by close examination of lashes and lid margins with slit lamp in order to identify the lice and nits [5, 8, 12, 14–18].

Recommended treatment options for phthiriasis palpebrarum are mechanical removal with forceps [5, 9], trimming or plucking of eye lashes [3, 4, 15], traumatic amputation [10], cryotherapy [19], argon laser photocoagulation [20], fluorescein eye drops 20% [21], physostigmine 0.25% [12, 22], lindane 1% [23, 24], petroleum jelly [24], yellow mercuric oxide ointment 1% [16], malathion drops 1% or malathion shampoo 1% [2, 24], 1% gamma-benzene hexachloride cream [25], pyrethrin ointment [14], permethrin 1% cream [15], kerosene [11], and oral ivermectin [15, 26] and pilocarpine gel 4% [7, 13, 27]. None of the pediculicides are 100% ovicidal; manual removal of nits after treatment with any product is recommended.

In addition, family members, sexual contacts, and close companions should be examined and treated appropriately; clothing, towels, and bedding used by the patient within two to three days before treatment began should be machine washed (with water at least 55°C, 30 minutes) and dried on the hot cycle for 5–10 minutes. Items that cannot be washed can be dry cleaned or stored in a sealed plastic bag for two weeks [7–12, 15–18].

Our patient was treated successfully by sequential application of petrolatum jelly and 1% permethrin shampoo. Although these treatment options may be used alone, they were applied together to increase efficacy and to reduce the likelihood of recurrence. With this treatment regimen, numerous parasites of the eyelashes were destroyed within 3 days. No louse or nit was seen at the first week and in the follow-up examination at 2 months.

Petrolatum jelly (Vaseline) is made from waxy petroleum material that formed on oil rigs and distilling it. Its mechanism of action is still unknown. However, it covers the lice, probably closes breathing holes, and prevents their respiration or moving [24, 28]. It is not also ovicidal [14]. Permethrin is a synthetic compound based on the insecticidal components of naturally occurring permethrins and is used to treat head lice and crab lice. It kills both live lice and hatched lice (eggs), but not unhatched eggs; because of its lack of percutaneous absorption, toxicity is not a consideration [6, 28]. It is almost 100% pediculicide and 20 to 70% ovicide [14].

*In conclusion*, phthiriasis palpebrarum is commonly misdiagnosed as bacterial, viral, allergic conjunctivitis or seborrheic blepharoconjunctivitis. Careful slit-lamp examination will usually indicate the correct diagnosis and appropriate treatment. This report demonstrated that the petrolatum jelly and 1% permethrin shampoo used together were simple, cheap, safe, and effective treatment for phthiriasis palpebrarum and crab louse.

## References

[1] Nuttall G. H. F. (1918). The biology of *Phthirus pubis*. *Parasitology*.

[2] Rundle P. A., Hughes D. S. (1993). Phthirus pubis infestation of the eyelids. *British Journal of Ophthalmology*.

[3] Turow V. D. (1995). Phthiriasis palpebrarum: an unusual course of blepharitis. *Archives of Pediatrics and Adolescent Medicine*.

[4] Mansour A. M. (2000). Photo essay: phthiriasis palpebrarum. *Archives of Ophthalmology*.

[5] Yoon K.-C., Park H.-Y., Seo M.-S., Park Y.-G. (2003). Mechanical treatment of phthiriasis palpebrarum. *Korean Journal of Ophthalmology*.

[6] Jacobs S. B. Entomological Notes, Human Lice. http://www.ento.psu.edu/extension/factsheets/human-lice.

[7] Kumar N., Dong B., Jenkins C. (2003). Pubic lice effectively treated with Pilogel. *Eye*.

[8] Vijayalekshmi S. (2012). Phthiriasis palpebrarum. *Our Dermatology Online*.

[9] Lin Y.-C., Kao S.-C., Kau H.-C., Hsu W.-M., Tsai C.-C. (2002). Phthiriasis palpebrarum: an unusual blepharoconjunctivitis. *Zhonghua Yi Xue Za Zhi*.

[10] Burgess I. F. (1995). Human lice and their management. *Advances in Parasitology*.

[11] Gokhale A. M., Gokhale S. A. (1983). Kerosene application for pediculosis. *Indian Journal of Ophthalmology*.

[12] Couch J. M., Green W. R., Hirst L. W., De La Cruz Z. C. (1982). Diagnosing and treating Phthirus pubis palpebrarum. *Survey of Ophthalmology*.

[13] Turgut B., Kurt J., Çatak O., Demir T. (2009). Phthriasis palpebrarum mimicking lid eczema and blepharitis. *Journal of Ophthalmology*.

[14] Klaus S., Shvil Y., Mumcuoglu K. Y. (1994). Generalized infestation of a 3 1/2-year-old girl with the pubic louse. *Pediatric Dermatology*.

[15] Kiran B., Kareem S. A., Illamani V., Chitralekha S. (2012). Case of *Phthiriasis palpebrarum* with blepheroconjunctivitis. *Indian Journal of Medical Microbiology*.

[16] Ashkenazi I., Desatnik H. R., Abraham F. A. (1991). Yellow mercuric oxide: a treatment of choice for phthiriasis palpebrarum. *British Journal of Ophthalmology*.

[17] Ikeda N., Nomoto H., Hayasaka S., Nagaki Y. (2003). Phthirus pubis infestation of the eyelashes and scalp hairs in a girl. *Pediatric Dermatology*.

[18] Ngai J. W. S., Yuen H. K. L., Li F. C. H. (2008). An unusual case of eye itchiness. *Hong Kong Medical Journal*.

[19] Awan K. J. (1977). Cryotherapy in phthiriasis palpebrarum. *American Journal of Ophthalmology*.

[20] Awan K. J. (1986). Argon laser phototherapy of *Phthiriasis palpebrarum*. *Ophthalmic Surgery*.

[21] Mathew M., D'Souza P., Mehta D. K. (1982). A new treatment of phthiriasis palpebrarum. *Annals of Ophthalmology*.

[22] Cogan D. G., Grant W. M. (1949). Treatment of pediculosis ciliaris with anticholinesterase agents. *Archives of Ophthalmology*.

[23] Kincaid M. C. (1983). Phthirus pubis infesttation of the lashes. *Journal of the American Medical Association*.

[24] Burns A. D. (1987). The treatment of *Pthirus pubis* infestation of the eyelashes. *British Journal of Dermatology*.

[25] Solomon L. M., Fahrner L., West D. P. (1977). Gamma benzene hexachloride toxicity. A review. *Archives of Dermatology*.

[26] Burkhart C. N., Burkhart C. G. (2000). Oral ivermectin therapy for phthiriasis palpebrum. *Archives of Ophthalmology*.

[27] Pinckney J., Cole P., Vadapalli S. P., Rosen T. (2008). Phthiriasis palpebrarum: a common culprit with uncommon presentation. *Dermatology Online Journal*.

[28] Elgart M. L. (1999). Current treatments for scabies and pediculosis. *Skin Therapy Letter*.

